# Downregulation of S100 calcium binding protein A12 inhibits the growth of glioma cells

**DOI:** 10.1186/s12885-020-06768-7

**Published:** 2020-03-30

**Authors:** Chunhe Lu, Jia Liu, Mingze Yao, Lun Li, Guangyu Li

**Affiliations:** 1 Department of Neurosurgery, The First Affiliated Hospital of China Medical University, 155 Nanjing North Street, Heping District, Shenyang City, Liaoning Province P.R. China 110001; 2 Department of Neurosurgery, Anshan Hospital of the First Hospital of China Medical University, No.166,Minzhu Street,Tiexi District, Anshan, Liaoning Province P.R. China 110001

**Keywords:** S100A12, Glioma, Tumor, EMT, Apoptosis

## Abstract

**Background:**

S100 calcium binding protein A12 (S100A12) is a member of the S100 protein family and is widely expressed in neutrophil and low expressed in lymphocytes and monocyte. However, the role of S100A12 in glioma has not yet been identified.

**Methods:**

In the present study, we carried out immunohistochemical investigation of S100A12 in 81 glioma tissues to determine the expression of S100A12 in glioma cells, and evaluate the clinical significance of S100A12 in glioma patients. Futher we knockdown the S100A12 by shRNA, and evaluated cell proliferation, cell migration and cell apoptosis by MTT, colony formation assay, transwell assay,flow cytometry assa and western blot.

**Results:**

We found that S100A12 was upregulated in tissues of glioma patients and the expression was correlated to WHO stage and tumor size. Further, we found that knockdown S100A12 inhibits the proliferation, migration and invasion of glioma cells through regulating cell apoptosis and EMT.

**Conclusion:**

S100A12 plays a vital role in glioma progression, and may be an important regulatory molecule for biological behaviors of glioma cell lines.

## Background

Gliomas are the most common tumor in central nervous systems in adults, and that were divided to high grade glioma (Grades III and IV) and low grade glioma (Grades I and II) by the World Health Organization (WHO) [1]. The glioblastoma is belonged to the high grade glioma. The current standard of care for patient of glioblastoma followed by operation, radiotherapy, chemotherapy, and the prognosis was poor [2].

In the 2016 world health organization of tumors of the central nervous system, the authors used molecular parameters in addition to histology to define the glioma for the first time [3]. The previous abundant dedicated research into the molecular biology of gliomas has caused a rapid acceleration of the discovery of some of the key molecular mechanisms as well as the genetic and epigenetic underpinnings of these tumors [4]. Despite many advances in medical treatments such as surgery, radiotherapy and chemotherapy, the prognosis of glioma is still poor especially in glioblastoma with a median survival of 14.6 months [5]. Thus, identification of novel mechanisms to suggest new possibilities for treatment of glioma is urgently needed.

The S100 protein family belongs to inflammatory molecules subgroup which predominantly comprises calcium-binding proteins [6]. S100 protein has been reported to be participated in a series of pathological progresses, such as chronic inflammation, autoimmune diseases and malignancies [7]. S100A12 is a member of this family and is widely expressed in neutrophil and low expressed in lymphocytes and monocyte [8]. S100A12 has also been found to be involved in multiple cancers. S100A12 is close linked to inflammation and vascular invasion and contribute to cancer metastasis [9]. It has been reported that high levels of S100A12 has been correlated with good prognosis for patients with oropharyngeal squamous cell carcinoma [10]. In addition, S100A12 was significantly increased in colorectal cancer samples when compared adjacent normal colon tissues [11]. It has been reported that the expression of S100A4 is highly correlated with the progression of glioma which indicated that S100A4 plays a vital role in the pathogenesis of glioma [12]. However, the role of S100A12 in glioma hasn’t been fully illuminated. Therefore, the aim of this study was to explore the role of S100A12 in glioma. We assumed that S100A12 was upregulated in glioma tissues, and that knockdown of S100A12 resulted in a repression of apoptosis and a elevation of proliferation of glioma cells.

## Methods

### Patients and specimens

The clinical specimens were collected from glioma patients at the First Affiliated Hospital of China Medical University. Normal brain tissues were obtained from patients suffering from cerebral injury who were underwent internal decompression. Thyroid tissue from patients with thyroiditis was used as a positive control group because the protein expression of S100A12 had been well reported in thyroiditis. The patients were written consent and with the approval of the ethics committee of the China Medical University. Glioma samples were immediately fresh frozen in liquid nitrogen and then stored at − 80 °C until further analysis.

### Cell lines

A172, U373, U118, U251 and U87 were obtained from Chinese Academy of Sciences (Shanghai, China). The cells were cultured with Modifed Eagle’s Medium (DMEM), supplemented with 10% fetal bovine serum and antibiotics (100 U/ml penicillin, 100 mg/ml streptomycin). The cell lines were incubated in 37 °C, 5% CO2 saturation.

### Immunohistochemistry (IHC)

The glioma specimens and normal brain tissues were embedded in paraffin and then sliced to 4 μm sections. Rabbit polyclonal anti-S100A12 (1:200; Abcam, Cambridge, UK) and biotinylated goat anti-rabbit immunoglobulin G were used as primary and secondary antibodies. Then counterstaining with diaminobenzidine, sections were inspected under microscope.

### S100A12 knockdown

The shRNA vectors were purchased from GeneChem Company (Shanghai,China). The S100A12#1 sequence was 5′-CGACTTTCAAGAATTCATA-3′,the S100A12#2 sequence was 5′- GGATGCTAATCAAGATGAA − 3′ and the shRNA control (shNC) sequence was 5′- TTCTCCGAACGTGTCACGT − 3′.

### Cell proliferation analyses

The cell viability was determined by MTT assay. Cells were seeded on 96-well plates at 1 d, 2 d and 3d after transfection, then incubated in the medium containing 100 mg/0.1 ml of MTT (Sigma Aldrich) for 4 h and incubated at 37 °C. After removing the medium, the blue crystal layer attached to the surface of the material was dissolved using dimethyl sulfoxide (DMSO). The OD value was measured at 490 nm by enzyme immunoassay instrument.

### Colony formation assay

Cells were seeded into 6 well plates at a density of 500 cells/well and cultured at 37 °C,5% CO2 for 14 days. Cells were then fixed in 4% paraformaldehyde and stained with crystal violet solution. Colonies from 3 independent groups were counted and the data were presented as mean ± standard deviation (SD).

### Cell invasion and migration assays

Transwell assay was used to evaluate the cell migration and invasion of glioma cell lines. For migration assay, 5 × 10^4^ cells were seeded in the upper chamber with serum-free culture medium (200 μl), and the lower chamber was filled with 10% FBS medium. After culturing for 24 h, the cells were fixed with 4% paraformaldehyde and stained with gemsa for 15 min. The images were acquired under microscope and migrated cells were counted in 3 random fields. The method of invasion assay was similar to the migration analysis, while the upper chamber was coated with matrigel (BD Biosciences, San Jose, CA).

### Flow cytometry assay

After 72 h of transfection, cells were collected and subjected to flow cytometry. Cell apoptosis was quantifed using Annexin V-fluorescein isothiocyanate apoptosis detection kit I (BD Biosciences, San Jose, CA, USA). Cell apoptosis analysis was performed using a Flow Cytometry System (BD Bioscience, Bedford, MA, USA).

### Western blot analysis

Proteins from glioma cells were homogenized in cold PBS containing 0.05% Triton X-100 and protease inhibitor cocktail (Sigma-Aldrich, St. Louis, MO, USA). Protein samples were electrophoresed on 10% sodium dodecyl sulfate-polyacrylamide gels (Sigma), transferred onto PVDF membranes according to standard protocols, and then blocked with 5% dried skimmed milk in TBST for at least 1 h. The membranes were incubated overnight at 4 °C with the following antibodies: anti-S100A12, anti-E-cadherin, anti-N-cadherin (1:1000, Abcam, Cambridge, MA, USA), anti-cleaved caspase 3, anti-Bcl2, anti-Bax (1:1000, Cell Signaling Technologies), and anti-GAPDH (1:1000, Abcam), and then incubated with horseradish peroxidase-conjugated secondary antibodies (1:1000, Abcam) at room temperature for 1 h after washing 3 times using TBST. Blots were washed 3 times again and developed using an enhanced chemiluminescence kit (Amersham Pharmacia Biotech). Immunoblot band quantification was calculated by means of a Bio-Rad calibrated densitometer (GS-800) using the vendor’s software (Bio-Rad Laboratories); GAPDH was used as an internal reference for analyses.

### Statistical analysis

Statistical analyses were performed using the SPSS version 17.0 and GraphPad Prism version 5.0. The comparisons among multiple groups were conducted by the one-way analysis of variance (ANOVA). Pearson correlation analysis was applied for correlation analysis. Kaplan Meier analysis was used to construct the survival curves of the high expression group and the low expression group, and log rank test was used to compare the survival differences between the groups. A value of *P* < 0.05 indicated that the difference was statistically significant, and a value of *P* < 0.01 showed that the statistics were of highly significant difference.

## Results

### S100A12 expression is elevated in glioma tissues and is correlated with poor prognosis

**Fig. 1:**
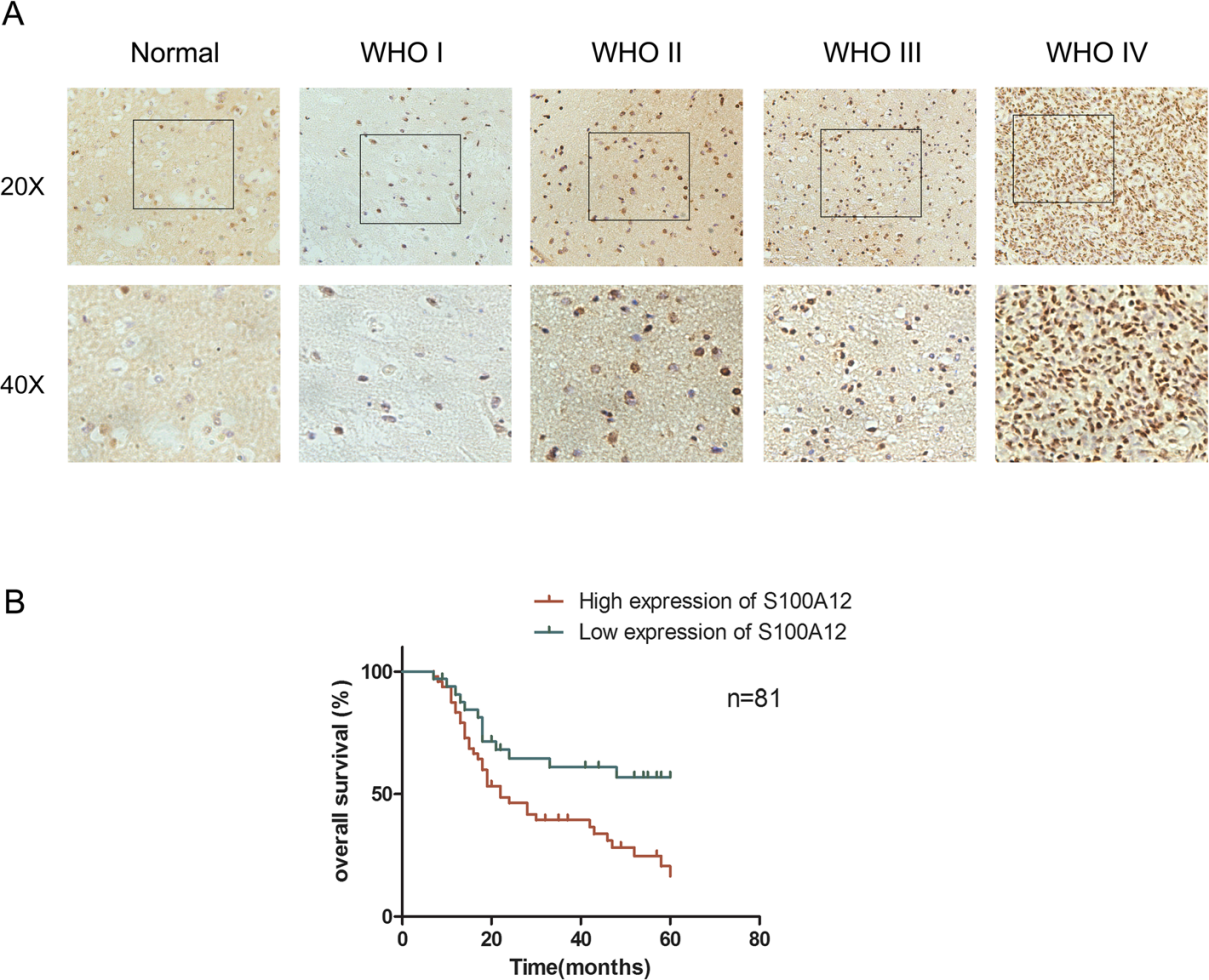
S100A12 expression in glioma and its associations with tumor progression. **a** The expression levels of S100A12 in different groups of the World Health Organization grade and normal tissues detected by IHC. **b** The significance of S100A12 expression level in overall survival

To investigate the expression of S100A12 in glioma individuals, we first analyze 81 cases of glioma tissues and 6 cases of control tissues. Of the samples, 48 and 33 samples showed high and low expression of S100A12 (Table 1). The immunohistochemistry results showed that S100A12 positive staining mainly occurs in the cellular compartment of nuclei, and the staining of glioma tissues was significantly stronger than normal brain tissues (Fig. 1a). Furthermore, the data from the survival analysis (Fig. 1b) indicated that the patients with high expression of S100A12 had shorter survival time than those with low expression.

**Table 1:** Correlation between S100A12 and clinic pathologic parameters of 81 glioma patients

Clinicopathologic parameters	Cases(N)	S100A12	expression	***P*** value
		Low	High	
Age (years)				
< 50	29	13	16	0.576
≥ 50	52	20	32	
Gender				
Male	35	13	22	0.565
Female	46	20	26	
Tumor siz (cm)				
< 2.5	31	19	12	0.003
≥ 2.5	50	14	36	
KPS				
< 80	34	17	17	0.149
≥ 80	47	16	31	
Grade				
I~II	24	17	7	0.003
III~IV	57	16	41

### S100A12 enhances glioma proliferation in vitro

**Fig. 2:**
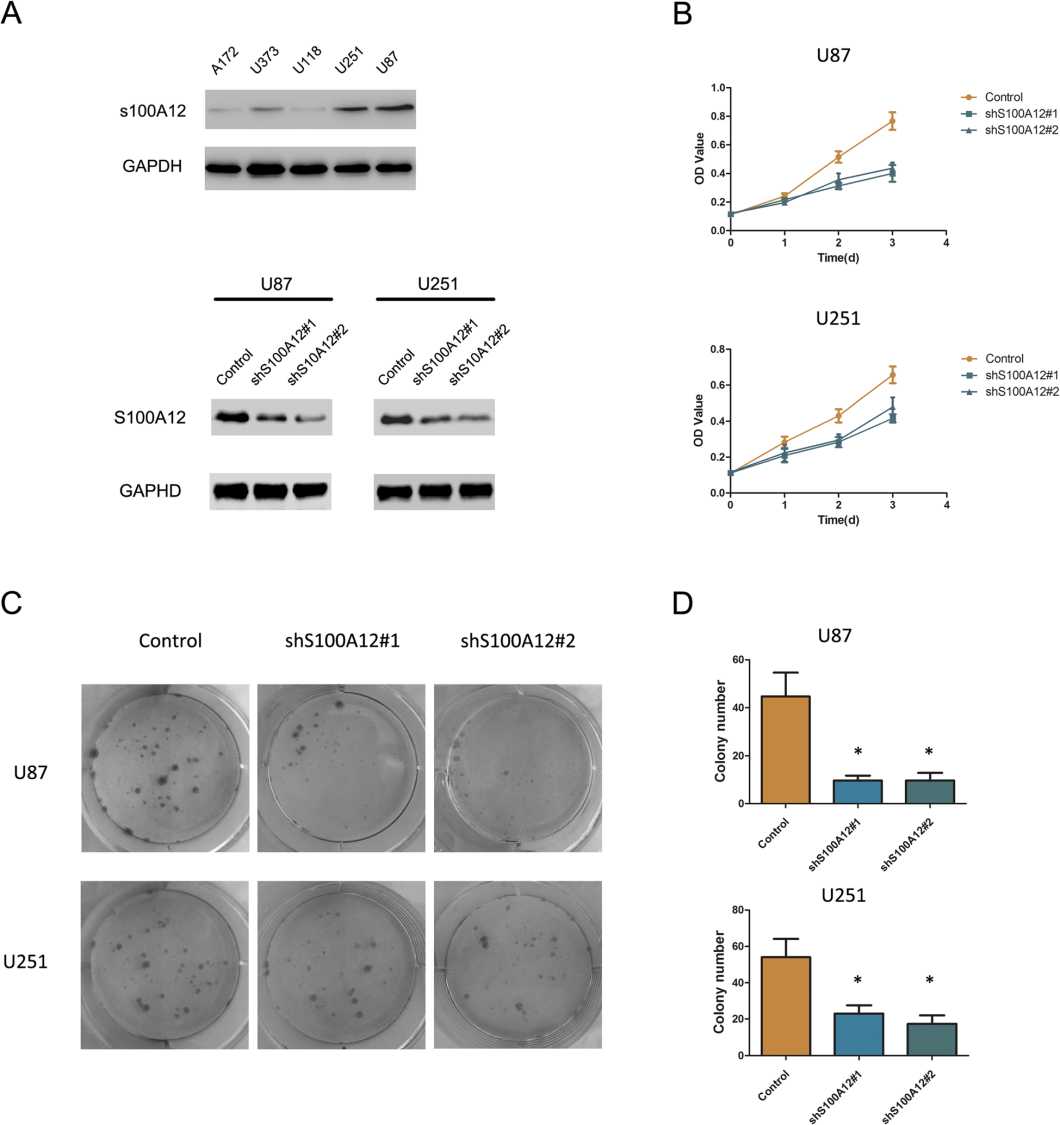
Knockdown of S100A12 inhibits glioma cells proliferation. **a** Western blot Western blotting analysis of S10A12 expression of A172, U373, U118, U251 and U87 cells and indicated glioma cells transfected with shS100A12#1, shS100A12#2 and control. **b** MTT assays revealed that of silencing S100A12 significantly decreased the growth rate of glioma cells. **c** and **d** Silencing S100A12 significantly reduced the colony formation ability of glioma cells in colony formation assay .* *P* < 0.05

To further analysis the biological behavior of S100A12 in glioma, we examined the expression of S100A12 by western blot analysis. We found that S100A12 expression was significantly up-regulated in U251 and U87 glioma cell lines compared with those in A172, U373 and U118 glioma cell lines (Fig. 2a). Then we stably depleted its expression in U251 and U87 glioma cells with lentivirus vectors (Fig. 2a). As determined by MTT assay, both shS100A12 in U251 and U87 showed a lower growth rate than those of control groups (Fig. 2b). Similar results were displayed in colony formation assay (*P* < 0.05) (Fig. 2c and d).

### S100A12 regulates glioma apoptosis in vitro

**Fig. 3:**
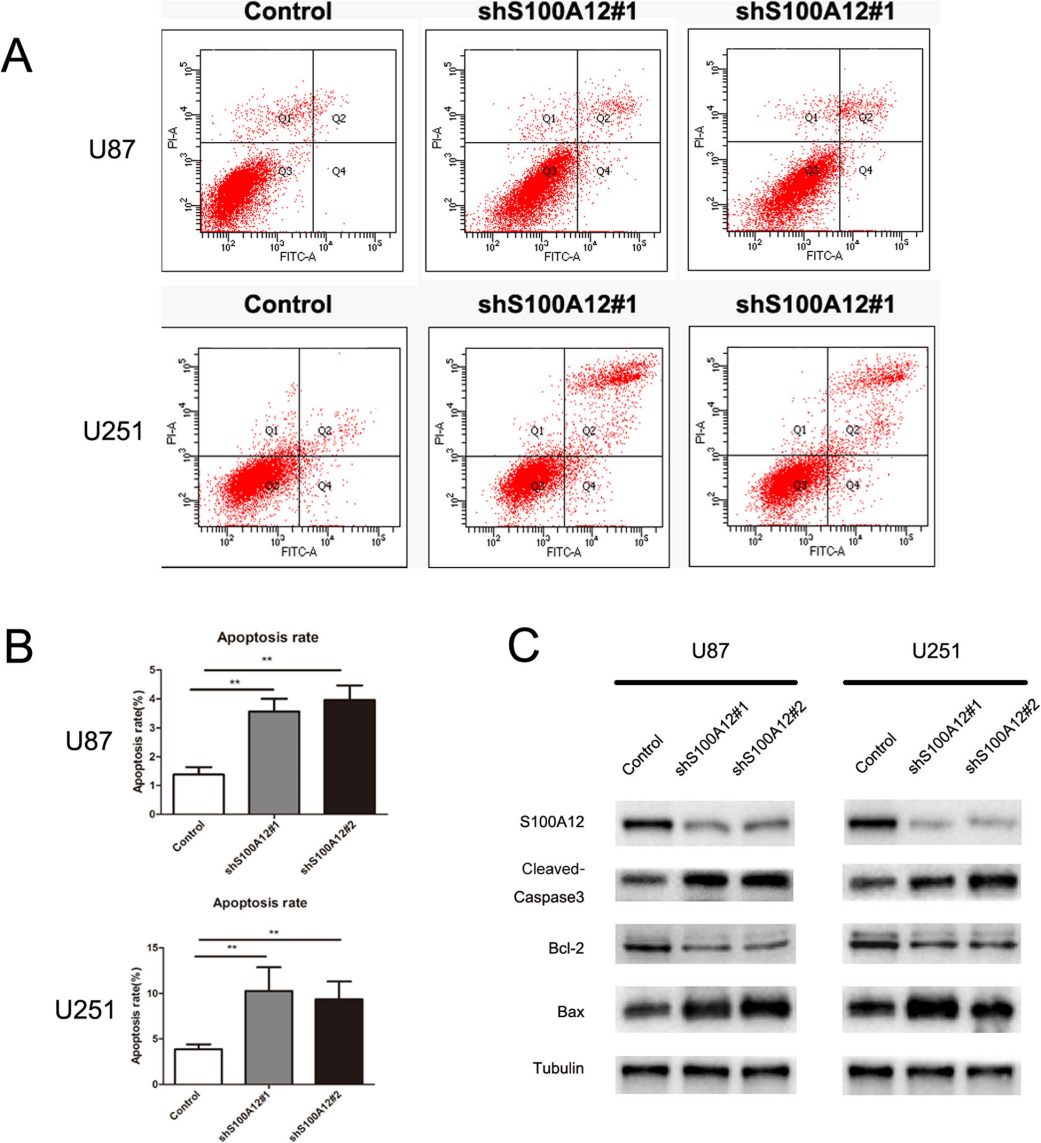
S100A12 knockdown induced glioma cells apoptosis and altered protein expression of Cleaved Caspase3, BCL2 and BAX. **a** and **b** The proportion of apoptotic PTC cells was increased upon S100A12 knockdown, as assessed by flow cytometry. ** *P* < 0.01. **c** knockdown of S100A12, downregulated the expression of BCL2 protein,and unregulated the expression of Cleaved Caspase3 protein and BAX

We then assessed the effect of S100A12 on the apoptosis of glioma cell lines by flow cytometry assay. The results showed the silence of S100A12 dramatically increased apoptosis rates in both U251 and U87 glioma cells (*P* < 0.01) (Fig. 3a and b). Consistently, Western blot assay also indicated that the protein levels of Cleaved-Caspase 3 and Bax increased when S100A12 knockdown in U251 and U87 cells, while Bcl-2 decreased (Fig. 3c). Taken together, S100A12 induced apoptosis in glioma cell.

### S100A12 increased the invasion and migration of glioma cells

**Fig. 4:**
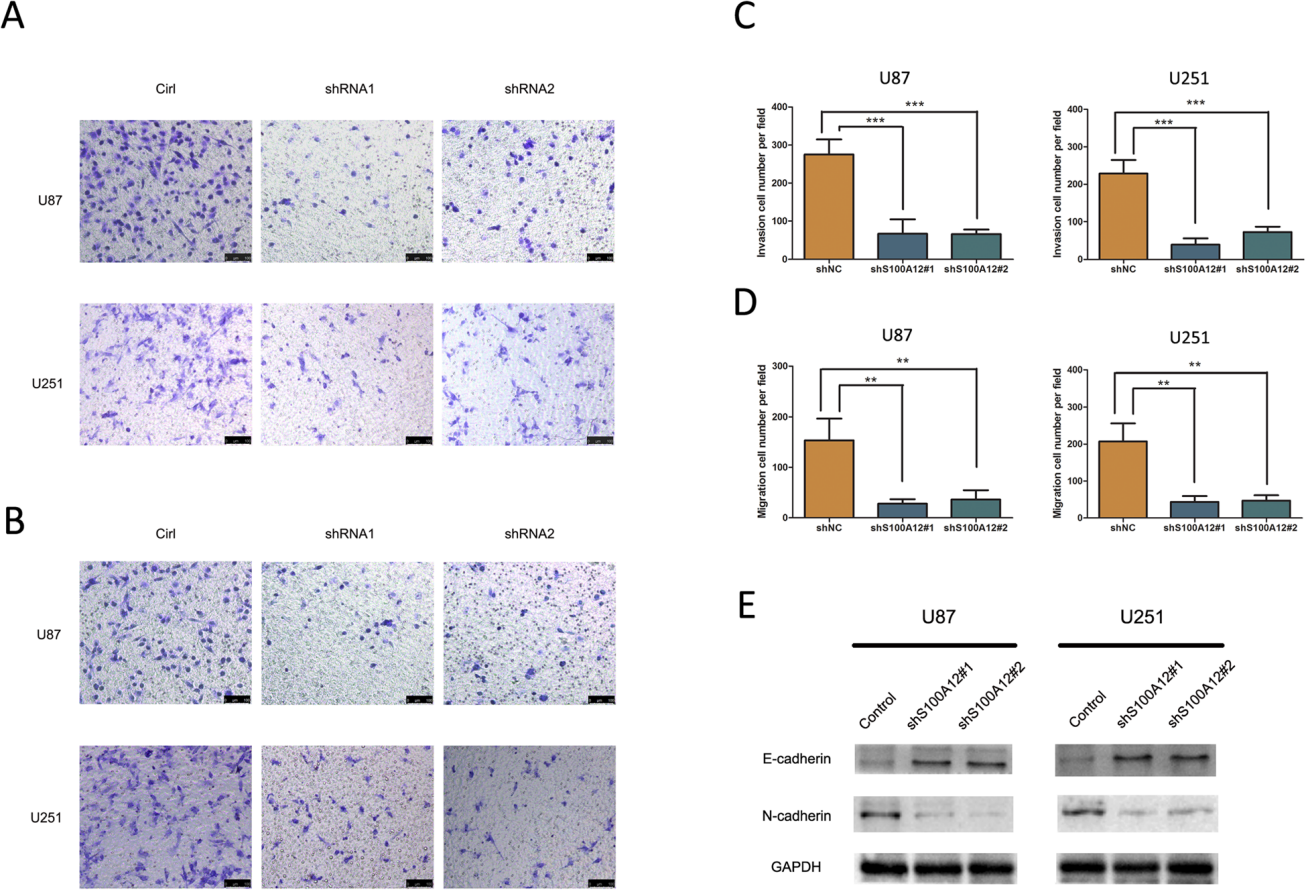
Effects of S100A12 knockdown on cell migration and invasion in vitro. **a** and **c** The invasive capacity was signifcantly impaired upon S100A12 knockdown in PTC cells, as examined by transwell invasion assay. **b** and **d** The migration capacity was signifcantly impaired upon S100A12 knockdown in PTC cells, as examined by transwell migraion assay. **e** Western blotting assay was performed to measure the E-cadherin and N-cadherin protein levels in PTC cells after knockdown of S100A12

We also investigated the effect of S100A12 on glioma cells invasion and migration. As shown in Fig. 4a-d, the transwell assay displayed that the invaded and migrated U251 and U87 cells were much fewer in the shRNA S100A12 groups than in the control group (*P* < 0.01).

The epithelial-mesenchymal transition (EMT) was analyzed by detecting the protein expression of E-cadherin and N-cadherin. Western blot assay showed that the protein levels of E-cadherin increased after S100A12 knockdown in U251 and U87 cells, while the protein levels of N-cadherin decreased when silencing of S100A12 (Fig. 4e). Taken together, S100A12 increased the invasion and migration of glioma cells and reversed the EMT phenotype in glioma cells.

## Discussion

Glioma is the most common central nervous system tumor and is featured by high malignancy and recurrence [1]. Although earlier studies have found novel treatment for glioma, the prognosis of this tumor remains poor [13, 14]. Thus, verification of potent therapeutic target is essential to the diagnosis and treatment of glioma.

In this study, we found that the protein expression of S100A12 was significantly increased in human glioma samples and the overexpression of S100A12 may be closely correlated with the prognosis of glioma patients. In addition, silencing of S100A12 markedly reduced the proliferation and the EMT process, whereas increased the apoptosis in two glioma cell lines, U87 and U251.

The S100 protein family has been reported in the regulation of many pathological processes, such as cell proliferation, migration and cell cycle advancement [15]. Of this subfamily, S100A12 is participated in the pathogenesis of multiple cancer-related diseases [16]. To the best of our knowledge, this study is the first to examine the expression of S100A12 in glioma patients. In this study, we found that the protein expression of S100A12 was significantly up-regulated in human glioma samples. Our study also examined the correlation between the expression of S100A12 with pathological characteristics. Our results showed that the overexpression of S100A12 was closely related with poor prognosis of glioma patients. These results indicated that S100A12 might contribute to the progression of glioma. The present study was in line with the earlier study on the expression of S100A12, which was highly correlated with the prognosis and high survival rates of oropharyngeal squamous cell carcinoma patients [10]. Huang et al. has also demonstrated that S100A12 might be serve as a new prognostic target to examine the recurrence and progression of the early stage of hepatectomy [17]. These results suggested that S100A12 might act as a biomarker in the progression of glioma patients.

In this study, silencing of S100A12 significantly down-regulated the cell viability and proliferation in two glioma cell lines. Furthermore, knockdown of S100A12 participated in the glioma cell apoptosis, which significantly increased the percentage of apoptosis rates in U87 and U251 cell lines. Moreover, silencing of S100A12 also caused the up-regulation of the protein expression of cleaved caspase 3 and Bax and down-regulation of Bcl2.These results indicated that S100A12 might contribute to the proliferation and apoptosis of glioma cells.

EMT is a process associated with the invasion and metastasis of cancer, and consequently enhances the metastasis of tumor [18]. Reduced expression of epithelial biomarker E-cadherin and elevated expression of mesenchymal biomarker N-cadherin are the most principal characteristic of EMT [19]. Cancer cells in EMT phenotype resulted in tumor metastasis and poor prognosis in patients [20]. It has been proved that glioma underwent EMT presented increased invasion and cancer metastasis, suggesting that EMT contributes to the progression of glioma [21–23]. Our results discovered that silencing of S100A12 dramatically increased the expression of E-cadherin and reduced the expression of vimentin and N-cadherin in U87 and U251 cells. These findings indicate that knockdown of S100A12 may suppress tumor progression by inhibiting the EMT process in glioma cells.

There were also some limitations in our study. We did not overexpress S100A12 in the cells, and did not test the effect of overexpression S100A12 on cell invasion, migration and apoptosis. In addition, the mechanism of the effect of S100A12 on glioma was not deep enough. For example, which protein does S100A12 specifically act on the EMT pathway.

## Conclusion

Our results give novel prospect that S100A12 plays a vital role in glioma progression. S100A12 was up-regulated in glioma samples and might contributed to the prognosis of glioma patients. Moreover, silencing of S100A12 inhibits the proliferation and EMT process and promotes the apoptosis of two glioma cell lines.

## Data Availability

The datasets used and/or analyzed during the current study are available from the corresponding author on reasonable request.

## Abbreviations

S100A12: S100 Calcium Binding Protein A12
shRNA: Short hairpin RNA
EMT: Epithelial-mesenchymal transition
PTC: Papillary thyroid carcinoma
WHO: World Health Organization
IHC: Immunohistochemistry
